# Detecting Dye-Contaminated Vegetables Using Low-Field NMR Relaxometry

**DOI:** 10.3390/foods10092232

**Published:** 2021-09-21

**Authors:** Sumaiya Shomaji, Naren Vikram Raj Masna, David Ariando, Shubhra Deb Paul, Kelsey Horace-Herron, Domenic Forte, Soumyajit Mandal, Swarup Bhunia

**Affiliations:** Department of Electrical and Computer Engineering, University of Florida, 216 Larsen Hall, P.O. Box 116200, Gainesville, FL 32611, USA; nmasna@ufl.edu (N.V.R.M.); dariando@ufl.edu (D.A.); shubhra.paul@ufl.edu (S.D.P.); khoraceherron@ufl.edu (K.H.-H.); dforte@ece.ufl.edu (D.F.); soumyajit@ece.ufl.edu (S.M.); swarup@ece.ufl.edu (S.B.)

**Keywords:** food adulteration, dye additives, nuclear magnetic resonance, relaxometry

## Abstract

Dyeing vegetables with harmful compounds has become an alarming public health issue over the past few years. Excessive consumption of these dyed vegetables can cause severe health hazards, including cancer. Copper sulfate, malachite green, and Sudan red are some of the non-food-grade dyes widely used on vegetables by untrusted entities in the food supply chain to make them look fresh and vibrant. In this study, the presence and quantity of dye-based adulteration in vegetables are determined by applying ^1^H-nuclear magnetic resonance (NMR) relaxometry. The proposed technique was validated by treating some vegetables in-house with different dyes and then soaking them in various solvents. The resulting solutions were collected and analyzed using NMR relaxometry. Specifically, the effective transverse relaxation time constant, *T*_2,*eff*_, of each solution was estimated using a Carr–Purcell–Meiboom–Gill (CPMG) pulse sequence. Finally, the estimated time constants (i.e., measured signatures) were compared with a library of existing *T*_2,*eff*_ data to detect and quantify the presence of unwanted dyes. The latter consists of data-driven models of transverse decay times for various concentrations of each water-soluble dye. The time required to analyze each sample using the proposed approach is dye-dependent but typically no longer than a few minutes. The analysis results can be used to generate warning flags if the detected dye concentrations violate widely accepted standards for food dyes. The proposed low-cost detection approach can be used in various stages of a produce supply chain, including consumer household.

## 1. Introduction

Food adulteration has reportedly increased over the last few years because of the complex supply chain of food from producer to consumer. Due to urbanization, consumers rely on growth, processing, transportation, and supply of food by multiple entities in the supply chain [1]. An untrusted entity can cause adulteration of food at any of these stages. Adulteration can take numerous forms, e.g., deliberate addition of substances with adverse health outcomes, not meeting desired product quality metrics, imitating other food substances, and using false labels on food packaging [2]. Human health is sensitive to food and thus can be affected by acute or chronic exposure to adulterated products. Even major health hazards, involving liver, vision, skin, and stomach disorders, are directly associated to adulterated food intake [3]. Foods like vegetables, fruits, fish, or meat adulterated with formalin have been found to be responsible for asthma and cancer [3]. Use of chemical pesticides has been linked to severe health problems, such as nerve damage and cancer [4]. There is also evidence that dye additives are responsible for genotoxicity, hypersensitivity, and carcinogenicity [5].

**Figure 1:**
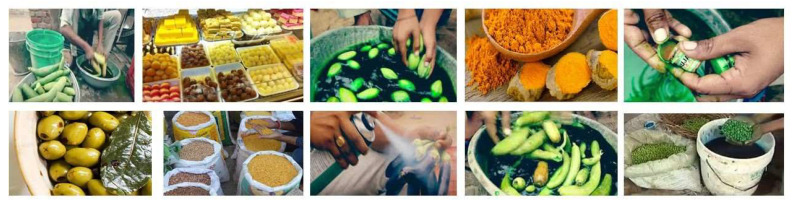
Various instances of vegetables and other consumables being adulterated with harmful chemicals. In most cases, cheap, industrial-grade dyes are used instead of food colors to maximize profits [11,12,13,14].

Synthetic dyes are added to many foods to provide them with a fresh look and compensate for natural color variations. These dyes are often harmful for the health and may even be carcinogenic [6]. Therefore, it is very important to understand the ingredients of food items before consuming them. This information is generally available for packaged foods since genuine product labels include the names of any dyes within the list of ingredients. However, fresh fruits and vegetables are generally not labeled. Dishonest entities in the food supply chain can exploit this lack of information to add toxic dyes to fruits and vegetables that make them appear fresh and vibrant to customers. Some real-world examples of this practice are shown in Figure 1. Existing methods to detect many of these dyes have been thoroughly reviewed in [6]. For example, chromatographic, physiochemical, sensory, spectroscopy, and DNA-based detection methods have been combined with chemometrics for a wide range of adulteration-detection applications [7]. Detection approaches that are particularly suitable for dyes include capillary electrophoresis, electrochemical voltametric analysis, and amperometry [6,7,8]. To illustrate, carcinogenic compounds, like malachite green [9] and Sudan red [10], can be easily detected by liquid chromatography, gas chromatography, capillary electrophoresis, amperometry, and plasmon resonance light scattering [6].

Traditional methods have shown promising results in detecting food dyes with very high accuracy [6]. However, they have some limitations. Firstly, they require a labor-intensive set of tasks that ranges from sample preparation to analysis. Therefore, the experiments require a large expenditure of time and human effort, making them unsuitable for at-home and field applications. Secondly, some of these methods often require expensive instrumentation that is often unavailable in the low- and middle-income countries where dye-based adulteration is most common [7]. For example, NMR spectroscopy requires highly uniform magnets, which are bulky and expensive [15,16]. Thirdly, low-cost methods generally detect adulteration by observing anomalies in basic physical or chemical properties of the suspect substance (e.g., viscosity, pH, or electrical conductivity) [6,7]. However, modern “smart” adulteration techniques can bypass such simple detection methods [17]. Therefore, to confront the food adulteration issues, i.e., the deliberate or accidental contamination of food items with banned substances, the food industry, government bodies, and consumers need sensitive, rapid, reliable, inexpensive, widely applicable, and difficult-to-attack methods to detect adulterated foods. Spectroscopy meets many of these criteria and is promising for detecting adulteration. During spectroscopy-based analysis, the chemical composition of a food product is investigated by measuring its frequency-dependent absorption or reflection spectra. Absorbance-based spectroscopy is mostly used for liquids, whereas reflection-based spectroscopy is used to identify fillers and adulterants, such as low-cost spices and dyes used to mask ageing. A variety of spectroscopic techniques, including near-infrared (IR), mid-IR, Raman, nuclear quadrupole resonance (NQR), and nuclear magnetic resonance (NMR), have been successfully used for monitoring food quality [18]. Each technique has its own advantages and disadvantages, which makes the optimum choice strongly application dependent.

NMR is rapidly emerging as an important analytical technique for food analysis and screening [19]. NMR-based methods can be grouped into three major measurement categories: imaging, spectroscopy, and relaxometry. NMR spectroscopy has many applications in food analysis and adulterant detection. For example, it has been used to detect Sudan red in paprika powder with higher sensitivity than Raman or IR spectroscopy [10]. Nevertheless, NMR is intrinsically a bulk measurement method, so detecting adulterants at extremely low concentrations (e.g., parts per billion) remains challenging [10]. Moreover, high-resolution NMR spectroscopy requires a strong and highly uniform static magnetic field (known as *B*_0_). Such fields are typically generated using large cryogenically cooled superconducting coils, thus resulting in very high installation and maintenance costs. A recent work proves that cryogen-free, desktop-sized permanent magnets can provide a lower-cost alternative [20]. Nevertheless, such magnets must be temperature-stabilized and manually-calibrated, so costs are still quite high (typically at least $20,000) [15,16]. As a result, complete NMR spectrometers (which combine the magnet with sample interrogation and readout electronics) cost $50,000 or more. Thus, there is a need for lower-cost alternatives for analyzing food samples.

NMR relaxometry provides such an alternative since it can be performed in a relatively weak and inhomogeneous *B*_0_ field, which in turn allows the size, complexity, and cost of the magnet to be greatly reduced [21,22]. Relaxometry focuses on measuring the nuclear spin relaxation times of specific substances present in a sample, namely the spin- lattice (*T*_1_) and spin-spin (*T*_2_) time constants; the translational diffusion coefficient (*D*) can also be measured. In a semi-classical picture, atomic nuclei with non-zero spin can be modeled as rotating magnetic dipoles. The static *B*_0_ field tends to align these dipoles (by convention, along the *z*-axis) much like compass needles in the Earth’s magnetic field, thus resulting in non-zero magnetization of the sample in thermal equilibrium. A second, time-varying magnetic field (known as *B*_1_) can be applied to perturb the magnetization away from equilibrium. Once *B*_1_ is removed, the sample gradually returns to equilibrium; this process is known as relaxation [23]. Specifically, *T*_1_ is the time constant for re-establishment of the equilibrium “longitudinal” magnetization, while *T*_2_ is the time constant for decay of the non-equilibrium “transverse” magnetization.

The two parameters are generally not equal to each other (in almost all cases, *T*_1_ ≥ *T*_2_) and also exhibit different dependencies on *B*_0_ field strength and temperature [21].

Several studies have used ^1^H-NMR and ^13^C-NMR NMR spectroscopy to detect food dyes (e.g., azo dyes) in solution [24,25]. Azo dyes are water-soluble, organic compounds that contain a functional group of the form R−N = N−R’, where R and R’ are typically aromatic groups. These dyes are widely used in some foods and also in the textile industry; common examples include Sudan red, metanil yellow, and malachite green. However, the NMR relaxation properties of aqueous solutions of azo dyes have not been carefully studied. This paper seeks to use the *T*_1_ and *T*_2_ relaxation time constants to detect these dies in food samples. To the best of our knowledge, it is the first to show that NMR relaxometry can be used for rapid and low-cost detection of multiple dyes (including malachite green and Sudan red) present within common vegetables.

NMR relaxometry can be used to determine the presence and quantity of a target compound with the help of a reference sample and chemometric analysis. In this approach, relaxometry was first performed on a reference sample and its relaxation time recorded. Next, relaxometry was performed on the test sample, and the relaxation time was again recorded. Finally, the relaxation times were compared to detect the presence and quantity of the target compound. Several methods, including linear regression, comparison with internal and external standards, and comparison of relaxation spectra, were used to quantitatively analyze the resulting data [26,27]. Linear or nonlinear regression on *T*_1_ and/or *T*_2_ values is simple to implement and numerically stable, while finding and preparing an appropriate reference compound (i.e., internal or external standard) is sometimes troublesome. However, both regression- and standards-based methods tend to fail for complex mixtures due to overlap between the *T*_1_ and/or *T*_2_ values of different components. Comparison of relaxation spectra generated using Laplace inversion is well-suited for such complex samples but suffers from limited resolution due to the numerically ill-conditioned nature of the inverse Laplace transform [27]. In this study, a simple and practical approach was developed for quantification of multiple food dyes by combining an external reference with nonlinear regression.

## 2. Materials and Methods

To simplify sample preparation, deionized (DI) water was used as the reference sample for all dyes, which is acceptable when only a single dye is present in a given test sample. The latter is a reasonable assumption since the goal of most dye-based adulteration is to impart a single color (e.g., green, orange, or red) to the vegetable or fruit in question. Finally, a general nonlinear regression method for quantitative analysis of the acquired relaxation data was used [10]. The details of this process are described next.

### 2.1. Dyes and Vegetables

A large number of chemical dyes have been used to make vegetables look fresh and vibrant [6], many of which are inedible and harmful to human health. For this study, three widely-used dyes were chosen: copper sulfate, malachite green, and Sudan red [28]. The first dye, copper (II) sulfate (CuSO_4_), is an inorganic compound that dissolves in water to produce a dark blueish-green solution. When dipped in this solution, green vegetables, like bitter gourds, peas, and cucumbers, turn dark or vibrant green. Unfortunately, CuSO_4_ is poisonous if ingested in large quantities (>1 gm) [29], with symptoms ranging from slight nausea to severe gastrointestinal infections and other diseases [28]. For this study, three different green vegetables, namely bitter gourd, okra, and pointed gourd (also known as parwal), were purchased from a local store and dyed using copper sulfate. The second dye, malachite green, is the monochloride salt of an aromatic cation (a triarylmethane) with formula C_23_H_25_N_2_^+^ [30]. It is generally used to color materials like leather or silk but because of its green hue is also illegally used to color vegetables, like peas and green chilies [28]. However, it is moderately toxic (even at concentrations as low as 0.1 µg/mL) and may also be carcinogenic [29]. In this study, yellow and green peas were dyed using malachite green. The third dye, Sudan red, is a reddish-orange lysochrome azo dye with formula C_17_H_14_N_2_O_2_ [31]. This chemical is known to be carcinogenic and banned in food items but nevertheless continues to be illegally used to color red chilies, red chili powder, red capsicum fruits, red pepper, chili jam, and tomatoes [32,33]. In this study, red chilies were dyed using Sudan red. All the dyes were purchased from Sigma-Aldrich (St. Louis, MO, USA), while the vegetables were obtained from local grocery stores (Gainesville, FL, USA).

### 2.2. NMR Relaxometry Instrumentation

**Figure 2:**
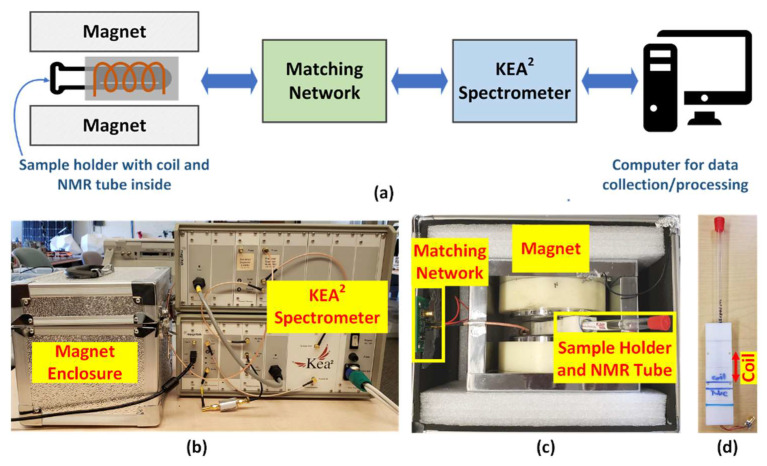
(**a**) A block diagram of the experimental setup; (**b**) a picture of the actual measurement setup; (**c**) an inside view of the magnet enclosure; and (**d**) a picture of the 3D-printed sample holder parts with the coil and an NMR tube inserted.

A block diagram of the overall experimental setup is illustrated in Figure 2a. The setup uses a benchtop permanent magnet (Spincore Technologies Inc., Gainesville, FL, USA) with a measured field strength of 0.5266 T at room temperature, resulting in a nominal ^1^H-NMR resonance frequency of 22.6 MHz. A 3D-printed holder containing the solenoid probe coil and NMR sample tube is centered between the magnetic poles [34]. The holder is coupled to a commercial benchtop NMR spectrometer (Kea^2^, Magritek Inc., Malvern, PA, USA) through a two-capacitor impedance matching network [35]. The spectrometer is powered by two 12-V, sealed lead-acid (SLA) batteries with a capacity of 18 Ah (not shown in the figure) and connected to a personal computer using a USB interface. A proprietary graphical user interface (GUI)-based software, Prospa, is used to control the spectrometer and acquire experimental data.

Figure 2b shows a photograph of the experimental setup. The permanent magnet, matching network, and sample holder are placed within a metallic enclosure that pro- vides electromagnetic shielding from external radio frequency (RF) interference by acting as a Faraday cage. Figure 2c shows the internal layout of this enclosure, while Figure 2d shows a more detailed view of the sample holder with a 10-mm thin-wall precision NMR tube (Wilmad-LabGlass, Vineland, NJ, USA) inserted into it.

The probe coil was hand-wound using AWG 22 copper wire. The signal-to-noise ratio (SNR) of the NMR measurements [36] was maximized by iteratively optimizing the coil geometry to maximize its quality factor (*Q*) at the ^1^H-NMR resonant frequency (*f*_0_ ≈ 22.6 MHz). The final design consisted of a tightly-packed solenoid with a relatively short length-to-diameter ratio (*L* ≈ 2 cm and *d* ≈ 10 mm, resulting in *L*/*d* ≈ 2) but a relatively large number of turns (*N* = 13). Coil properties around *f*_0_ were measured using a vector network analyzer (E5071C, Agilent Technologies). The results (inductance = 840 nH, series resistance = 415 mΩ) confirm adequately high quality factor (*Q* 287) and self-resonant frequency (*f_SRF_* ≈ 130 MHz). The estimated position of the coil within the sample holder is shown in Figure 2d.

### 2.3. Methodology

**Figure 3:**
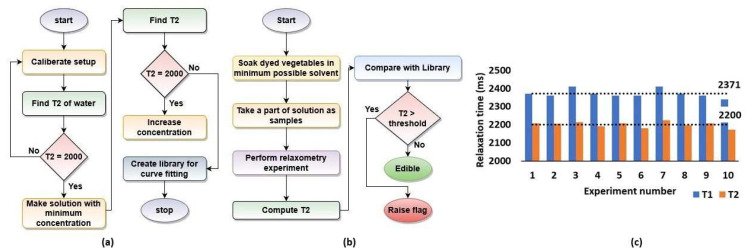
(**a**) Flowchart showing the process of creating a library; (**b**) flowchart showing the process of determining unknown concentrations; (**c**) measured variation of *T*_1_ and *T*_2_ values for the reference sample (DI water) over 10 experiments.

Instead of measuring the adulterant in situ, it was first washed out into solution. For this purpose, the sample (fruit or vegetable) was soaked in a solvent with known properties (e.g., DI water or brine) for a few minutes. The concentration of adulterant in the solvent was then measured using NMR relaxometry. This process has several advantages, including (i) eliminating the effect of sample heterogeneity from the *T*_1_ and *T*_2_ measurements and (ii) greatly simplifying sample preparation. The acquired relaxation data were further analyzed in two steps: (i) library creation (Figure 3a) and (ii) quantifying the concentration of adulterant (Figure 3b). Our current implementation of both steps focused on *T*_2_ since it can be rapidly and accurately measured using the well-known Carr–Purcell–Meiboom–Gill (CPMG) pulse sequence [37,38], but the procedure can be readily extended to include *T*_1_ data (e.g., from an inversion recovery (IR) pulse sequence).

## 3. Results and Discussion

### 3.1. Library Creation

Calibration was carried out by using IRand CPMG pulse sequences to measure the *T*_1_ and *T*_2_ values of the reference sample, which is typically 12 mL of DI water. The measured values are *T*_1_ ≈ 2370 ms and *T*_2_ ≈ 2200 ms at room temperature (see Figure 3c). This value of *T*_1_ ≈ 2400 ms is in good agreement with earlier studies [39], while *T*_2_ is similar to *T*_1_, as expected for water [40].

The next goal was to confirm that aqueous solutions of all three dyes under study exhibited *T*_2_ contrast, i.e., a reproducible dependence of *T*_2_ on dye concentration. For this, known quantities of each dye were dissolved in a fixed amount (100 mL) of reference sample (either DI water or 0.5% NaCl solution) to create a library of solutions. For convenience, a solution containing *x* gram of a particular dye was referred as “*x*% solution”. Next, 12 mL of each solution was placed in an NMR sample tube and analyzed using a CPMG pulse sequence. The measured relaxation time constant is denoted by *T*_2,*eff*_ to distinguish it from that of the reference sample (DI water). In each case, the CPMG echo spacing (*t_E_*) was kept small enough to ensure that molecular diffusion did not significantly affect the value of *T*_2,*eff*_ [37].

**Figure 4:**
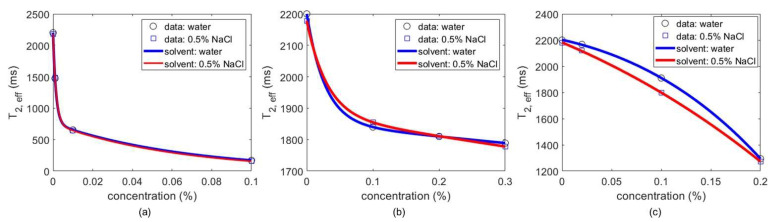
A library of vegetable dyes was created using the relationship between *T*_2,*eff*_ and its concentration. This library can be used to quantify the amount of dye used in vegetable adulteration. The libraries exhibiting these trends are shown for 3 different dyes: (**a**) copper sulfate, (**b**) malachite green, and (**c**) Sudan red. Separate library functions are shown for two different experimental solvents, namely DI water and 0.5% NaCl solution.

The smallest value of *x* (i.e., the sample weight) used within the proposed library was experimentally adjusted for each dye to ensure that the resulting change in *T*_2,*eff*_ could be accurately estimated within a few scans. For this, the measured CPMG echo decay curves were fit to mono-exponential functions of the form *Ae*^−*ntE/T*_2,*eff*_^ using least-squares function minimization; here, *A* is the initial signal amplitude, and *n* = 1, 2,... is the echo number. Figure 4 shows the measured dependence of *T*_2,*eff*_ on concentration for all three dyes. In each case, a monotonic decrease of *T*_2,*eff*_ with concentration was observed; the effect is particularly strong for CuSO_4_. As a result, sample concentration can be unambiguously estimated from the measured value of *T*_2,*eff*_.

The underlying cause for the observed decrease in *T*_2,*eff*_ with concentration is increased inter-molecular dipole-dipole (D-D) relaxation of the water molecules. Inter- molecular D-D relaxation is typically the dominant relaxation mechanism in dilute aqueous solutions [21]. It arises from time-varying fluctuations in the *B*_0_ field seen by each nucleus due to random thermal motion of other molecules or ions in the solution (which act like miniature dipole field sources). In the case of CuSO_4_, the effect is dominated by random motion of the added Cu2+ ions, which contain unpaired electrons and are thus paramagnetic [41]. In the case of the organic dyes, the effect is likely dominated by slower motion (and thus increased D-D relaxation rates) [21] of the loosely-organized shell of water molecules that surrounds each dye molecule due to mutual electrostatic attraction. Each shell is in rapid chemical exchange with bulk water molecules, thus explaining the observed mono-exponential echo decay curves.

The observed relationship between *T*_2,*eff*_ and concentration for each dye was quantified using nonlinear regression, i.e., least-squares curve fitting. The resulting functions can be inverted to estimate unknown dye concentrations, as described in the next section.

### 3.2. Detection of Unknown Concentrations

The calibration curves described in the previous section were used to estimate the concentration of dye washed out from adulterated vegetables. For this purpose, non- adulterated vegetables were purchased from a local market, dyed by immersing them in the appropriate solution, and air-dried to remove extra liquid. Finally, the adulterated vegetables were soaked in the reference solvent (typically DI water) to wash out the dye. The *T*_2,*eff*_ value of the solution was then analyzed using a CPMG pulse sequence.

**Figure 5:**
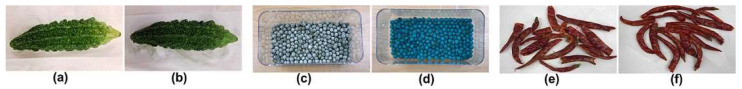
Comparison between the raw and dyed vegetables: (**a**) raw okra, (**b**) okra dyed with copper sulfate, (**c**) raw peas, (**d**) peas dyed with malachite green, (**e**) raw red chilies, and (**f**) red chilies dyed with Sudan red.

A careful set of experiments was performed to determine the optimum sample-preparation procedure. Firstly, the optimum solution concentration for dyeing vegetables was determined. Figure 4 shows that the NMR setup can reliably detect concentrations as low as 0.1–0.3%. Thus, a higher concentration (1%) was used to dye each vegetable. Specifically, 1% CuSO_4_ was used for pointed gourd, bitter gourd, and okra; 1% malachite green for peas; and 1% Sudan red for red dried chilies. The original (raw) and adulterated (dyed) vegetable samples are visually compared in Figure 5.

**Figure 6:**
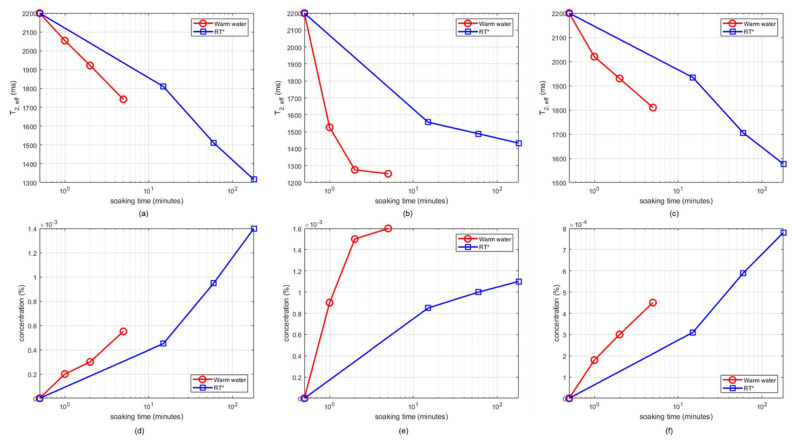
Estimated values of *T*_2,*eff*_ (top row) and concentration of extracted dye in solution (CuSO4, bottom row) as a function of time using water at room temperature and warm water at 60 °C: (**a**,**d**) pointed gourd; (**b**,**e**) bitter gourd; and (**c**,**f**) okra.

The vegetables were soaked in the corresponding dye solutions for 3 h and then air-dried for 12 h in room temperature. Then, it was determined the optimum combination of reference solvent, temperature, and soaking time, *t_soak_*, for washing out each dye. Firstly, both DI water and 0.5% NaCl solution were studied as reference solvents; the results were similar, so DI water was chosen for convenience. Secondly, the solvent temperature and soak time were varied. For water at room temperature, *t_soak_* = 5, 60, and 180 min were used. For warm water at 60 °C, *t_soak_* = 1, 2, and 5 min were used since the wash-out process (which is driven by diffusion) was expected to be significantly faster. Figure 6a–c show that *T*_2,*eff*_ values decreased with time as more dye (CuSO4 in this case) washed out into solution; the rate of change was significantly higher for warm water, as expected. Similarly, Figure 6d,f confirm that (i) the estimated dye concentrations increased with time, and (ii) warm water could extract most of the dye within *t_soak_* = 2 min, while much longer soak times were required at room temperature.

During the experiments, the optimized procedure described above (dyeing with 1% solution, drying for 12 h, soaking in warm water for 2 min, and finally estimating dye concentration from *T*_2,*eff*_ measurements) was repeated 10 times for each sample to ensure that the results are repeatable and consistent. The experiments confirm that both the presence of the chosen dyes and their extracted concentrations can be reliably estimated (with typical error < 4%) using the proposed technique.

### 3.3. Discussion

While the experiments in the paper were focused on three common dyes, the proposed method can be extended to any dye that exhibits NMR relaxation contrast (in *T*_1_ and/or *T*_2_) while in aqueous solution. Compounds containing paramagnetic ions (such as Cu^2+^ or Ni^2+^) fall into this category since they result in increased intermolecular D-D relaxation rates. Compounds with permanent electric dipole moments, such as most azo and aryl dyes, may also exhibit a small amount of relaxation contrast due to the reduced mobility of water molecules in their associated hydration shells. Additional relaxation contrast can be obtained by performing *T*_1_ measurements at different field strengths (e.g., by using an electromagnet to generate *B*_0_); this process is known as field-cycling relaxometry [42].

Besides generality, additional desirable features for the proposed food-adulteration detection platform include portability and cost-effectiveness. As noted earlier, NMR spectroscopy is expensive because of the need to generate a strong and highly uniform *B*_0_ field. While the magnet size and cost requirements can be significantly reduced by focusing on relaxometry, the large size and power consumption of the spectrometer electronics (which includes an analog front-end and a digital back-end) remains a barrier for portable and low-cost applications. Fortunately, recent work has demonstrated miniaturized and low-power versions of both the front- and back-ends. For example, a portable NMR spectrometer based on a custom front-end and a low-cost system-on-chip (SoC) back-end has been developed [43]. Such miniaturized and low-cost devices can be used to replace the benchtop spectrometer used in the current setup.

## 4. Conclusions

This paper has demonstrated, for the first time to our knowledge, a simple, low-cost, yet powerful technique that combines NMR relaxometry with nonlinear regression-based trend modeling to detect and quantify harmful dyes in vegetables. Our experimental results show that the proposed technique can reliably quantify the presence of three commonly used illegal dyes, namely copper sulfate, malachite green, and Sudan red, at concentrations as low as 1 g/L (0.1%). The proposed technique can be used for detecting and potentially quantifying chemical dye-based produce adulteration in various stages of a supply chain, including retail facilities and consumer households. Future work will focus on extending our approach to a wider range of chemical dyes and food items as well as further enhancing the detection sensitivity.

## Data Availability

Not applicable.
